# Responses of Phyllosphere Microbiome to Ozone Stress: Abundance, Community Compositions and Functions

**DOI:** 10.3390/microorganisms10040680

**Published:** 2022-03-22

**Authors:** Jiayu Liu, Manjiao Song, Xinyuan Wei, Huanzhen Zhang, Zhihui Bai, Xuliang Zhuang

**Affiliations:** 1 Beijing Key Laboratory of Water Resources and Environment Engineering, School of Water Resources and Environment, China University of Geosciences (Beijing), Beijing 100083, China; ljiayu@cugb.edu.cn (J.L.); huanzhen@cugb.edu.cn (H.Z.); 2 Key Laboratory of Environmental Biotechnology, Research Center for Eco-Environmental Sciences, Chinese Academy of Sciences, Beijing 100085, China; songmanjiao19@mails.ucas.ac.cn (M.S.); chaserxin@foxmail.com (X.W.); 3 Sino-Danish College, University of Chinese Academy of Sciences, Beijing 100049, China; 4 Xiongan Institute of Innovation, Xiongan New Area 071000, China; 5 Institute of Tibetan Plateau Research, Chinese Academy of Sciences, Beijing 100101, China

**Keywords:** phyllosphere microbiome, phylloremediation, ozone exposure, high-throughput sequencing, core taxa, microbial function

## Abstract

Ozone is a typical hazardous pollutant in Earth’s lower atmosphere, but the phyllosphere and its microbiome are promising for air pollution remediation. Despite research to explore the efficiency and mechanism of ozone phylloremediation, the response and role of the phyllosphere microbiome remains untouched. In this study, we exposed *Euonymus japonicus* to different ozone levels and revealed microbial successions and roles of the phyllosphere microbiome during the exposure. The low-level exposure (156 ± 20 ppb) induced limited response compared to other environmental factors. Fungi failed to sustain the community richness and diversity, despite the stable ITS concentration, while bacteria witnessed an abundance loss. We subsequently elevated the exposure level to 5000~10,000 ppb, which considerably deteriorated the bacterial and fungal diversity. Our results identified extremely tolerant species, including bacterial genera (*Curtobacterium*, *Marmoricola*, and *Microbacterium*) and fungal genera (*Cladosporium* and *Alternaria*). Compositional differences suggested that most core fungal taxa were related to plant diseases and biocontrol, and ozone exposure might intensify such antagonism, thus possibly influencing plant health and ozone remediation. This assumption was further evidenced in the functional predictions via a pathogen predominance. This study shed light on microbial responses to ozone exposure in the phyllosphere and enlightened the augmentation of ozone phylloremediation through the microbial role.

## 1. Introduction

Ozone is a typical atmospheric pollutant formed through photochemical reactions between volatile organic compounds (VOCs) and nitrogen oxide (NO_x_). The precursor gases emission is attributed to natural and anthropogenic sources, represented by plants, soils, vehicle exhaust and fossil fuels [1]. Ozone near the ground can directly deteriorate human health as a strong oxidant when reaching a specific concentration. Long-term exposure or inhalation will cause or exacerbate chronic respiratory disease and damage the respiratory system and lungs [2]. Additional harm to plants and agriculture has been stressed, including decreasing the photosynthesis rate [3], crop yield [4] and nitrogen metabolism [5]. In Beijing, ozone levels in 16 summer days (June, July, August) in 2021 exceeded the maximum daily 8-h average (160 μg·m^3^) set by Chinese standard GB3095-1996.

Plants have been considered natural sinks, presenting a prospect of sustainable ozone uptake. For example, the observed average ozone uptake was up to 11 nmol·s^−1^·m^−2^ of *Citrus* leaf [6]. Phytoremediation consists of nonstomatal adsorption and stomatal absorption. Before entering the stomata, ozone disappears once reacting in the gas phase or contacting external surfaces, but the process can be inhibited under dry conditions. Stomatal sink is the main contributor to ozone removal at the plant level, nevertheless leading to visible injuries [7]. The fate of ozone after entering the stomata remained unclear, nor is the efficiency and possible accumulative harm on the plant known [8].

Fortunately, plants are accompanied by massive microorganisms, especially those in direct contact with ozone, named phyllosphere microbiota. The phyllosphere includes the aerial plant surfaces [9], such as leaves, fruits, stems, etc. Up to 10^6^~10^7^ cells·cm^−2^ of bacteria [10] and massive fungi [11] were estimated to colonize the phyllosphere and might assist in ozone remediation. The known potential mechanisms comprise detoxification and plant growth promotion. Ozone poses toxicity via reactive oxygen species (ROS) [12], and bacteria with antioxidative properties can alleviate such stress by scavenging superoxide anion free radicals [13]. On the other hand, the negative impacts on plants may be due to a combination of direct ozone damage plus enhanced or diminished growth of pathogens or other plant-related microbes [14], i.e., the plant health and defense modulated by phyllosphere microbiota under ozone interference. However, the current knowledge of microbial responses to ozone stress and microbiome-ozone interactions remained largely untouched.

Previous studies focused on bacterial and archaeal communities’ changes in the rice rhizosphere in response to the elevated ozone level and suggested a reduction in diversity [15,16]. Given that the phyllosphere forms direct contact with ozone, [17] assessed the influences of ozone pollution on the phyllosphere bacteria by adopting the high-throughput sequencing (HTS) technology. The effects on phyllosphere fungi were also explored [18]. Nevertheless, the employed exposure intensity induced minor alterations, especially under the interference of other important determinants of the microbial community. Therefore, under the precondition of appropriate exposure intensity, investigating microbial responses and revealing core taxa with outstanding tolerance will significantly enhance the understanding and feasibility of ozone phylloremediation.

We chose a common species in the urban green belt, *Euonymus japonicus* (*E. japonicus*), as the experimental material in the present study. Before the exposure, we examined the removal efficiency of ozone in a quasi-sealed chamber. We conducted three levels of ozone exposure on *E. japonicus* and monitored the bacterial and fungal community in the phyllosphere via HTS and quantitative polymerase chain reaction (qPCR). Results above cast light on microbial responses to the ozone stress, mainly reflected by quantity, key taxa and function patterns.

## 2. Materials and Methods

*E. japonicus* was purchased from a flower market in Hebei province in August. Before the exposure, plants were cultivated in cylindrical pots (diameter: 30 cm, height: 20 cm) in a greenhouse used for the formal experiments for at least a week. They were provided with running water once a day and characterized by the tree canopy’s projected diameter (D) and the above-ground height (H), listed in Table S1.

The customized apparatus is illustrated in Figure S1. The “polluted” gas was generated from an ozone generator. Irregular chambers encircled by Teflon membranes were employed, with double side hung doors in the front, a solar fan in the corner and a hygrothermograph fixed on the sidewall. Tubes extended inwards linked to an ozone monitor to facilitate plant placement, gas mixing, monitoring of temperature (T) and relative humidity (RH) and ozone concentration monitoring, respectively. The specifications of the abovementioned components are shown in Table S2. Five 600-L chambers (two control groups, hereafter as CK, and three experimental groups) were placed in a greenhouse.

Five treatments were set up, including two chambers with soil and pot only and three with soil and potted *E. japonicus*. The pots were all covered with aluminum foil to avoid littering and material exchanges. The generated ozone was pumped in through silicon tubes at a constant rate of 1.2~1.4 L·min^−1^. Each circulation of the removal experiment lasted for 120 min with a sampling interval of 15 min. 

Five potted *E. japonicus* were placed in five chambers, among which two comprised the control group, with the remaining for ozone exposure. For low-level (L) exposure, the generated ozone was pumped in through silicon tubes at a constant rate of 0.8~1.2 L·min^−1^ from 10:00 to 16:00 daily. The inner ozone concentration was monitored at the interval of 1 h (Figure S2), sustaining the level at 156 ± 20 ppb, referring to the 1-h air quality standard (GB3095-1996) of 102 ppb (25 °C, 1 atm). The control chambers were introduced with the ambient air (32 ± 12 ppb) at the same rate. The corresponding T (°C) and RH (%) were synchronously recorded as Figure S3. 500 mL of running water was supplied daily. The exposure lasted 28 d, and samplings were conducted at 10 d and 28 d in triplicate. For high-level (H) exposure, the inner ozone level stabilized at 4575 ± 756 ppb for 3 d, with an average T and RH of 25 °C and 51%, and samplings were conducted at 0 d and 3 d in triplicate. For ultrahigh-level (U) exposure, the inner ozone level stabilized at 10,212 ± 894 ppb for 5 d, with an average daily T and RH of 30 °C and 45%, and samplings were conducted at 0 d and 5 d in triplicate.

The leaves were sampled from different layers, i.e., plant heights to avoid any interference. Details of the leaf sampling were described in [19], with the minor modification that each triplicate contained 40 g intact and healthy leaves. The eluant was filtered with sterile nylon films (0.22 µm × 50 mm) with the intercepted part for DNA extraction. DNA was extracted by Fast^®^DNA SPIN kits (MP Biomedicals, Santa Ana, CA, USA) following manufacturer’s instructions, quantified by NanoDrop ND2000 spectrophotometer (Thermo Scientific, USA) and checked by the agarose gel electrophoresis (1.0%) [20].

The primers consisted of 16S rDNA region (799F_1115R) [21] and ITS1 region (ITS1F_ITS2R) [22], Procedures of PCR analysis were previously described in detail [23].

DNA sequences analysis was performed using the free online platform of Majorbio Cloud Platform (www.majorbio.com (accessed on 15 March 2022)). Poor-quality sequences were filtered via FLASH software [24]. The upgraded sequences were clustered into operational taxonomic units (OTUs) with a similarity threshold of 97% on UPARSE [25], supplemented with taxonomic analysis based on SILVA database (Release138, [26]). Alpha diversities at the OTU level were calculated with the online mothur platform (v.1.30.2). The 16S taxonomic lineage was transformed based on the SILVA database [27] into the lineage of prokaryotes in the Kyoto Encyclopedia of Genes and Genomes (KEGG) database [28] to annotate bacterial functions in Tax4Fun. FUNGuild was applied for fungal annotation [29]. 

qPCR was employed as previously described [19]. Alpha diversity indexes were presented in average and standard deviation (*n* = 3), and *p* values were calculated by one-way ANOVA [30] followed by a LSD-test. To reduce complexity, only the 15 most abundant genera were included in differential species analysis, with a standard deviation (*n* = 3) and *p* values calculated by Mann–Whitney–Wilcoxon (MWW) test. *p* < 0.05 indicates statistical significance. The calculations and visualizations proceeded in R, SPSS 20.0, and Origin 2017.

## 3. Results

### 3.1. Ozone Phylloremediation

**Figure 1:**
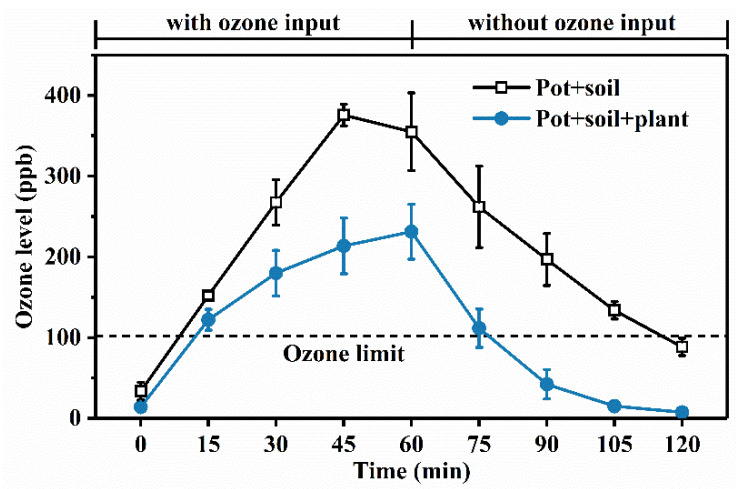
Time courses of ozone level within the sealed chambers. Treatments included pot with soil and pot, soil with E. japonicus, both covered with aluminum foil. The ozone generation lasted for 60 min, and error bars represent standard deviation (*n* = 3). The dashed line refers to the ozone concentration limit (GB3095-1996).

The phyllosphere of *E. japonicus* was employed for ozone remediation within the quasi-sealed chambers. The experiment lasted for 120 min with a sampling interval of 15 min. During the first 60-min of continuous ozone generation (Figure 1), the ozone concentration in the chambers without *E. japonicus* peaked at 375 ppb after 45 min and then fell to 88 ppb in the end. In the presence of *E. japonicus*, the ozone level peaked at 231 ppb after 60 min, dropped to the standard (102 ppb) after 15 min, and subsequently fell to the detection limit. The removal by the pot and soil mainly depends on physical deposition [6] to the surrounding membrane and the pot surface. Once including *E. japonicus*, physical deposition was greatly enhanced, with the stomata playing a critical role [7]. Plants were inclined to emit biogenic volatile organic compounds (BVOCs) to generate ozone [31], which was negligible within the short testing period. *E. japonicus* presented excellent ozone removal potential, facilitated by the plant and microbiome, but the respective contribution remains unclear.

### 3.2. Low-Level Ozone Exposure

**Figure 2:**
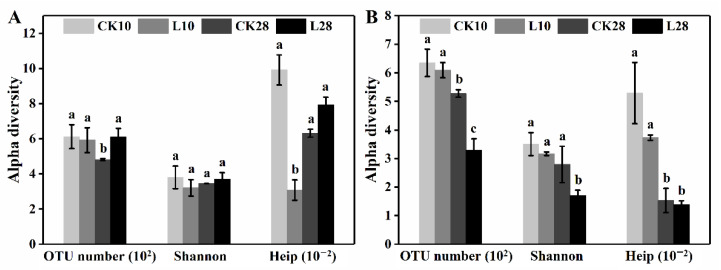
Alpha diversity changes of the bacterial (**A**) and fungal (**B**) community in the phyllosphere during the low-level ozone exposure. OTU number, Shannon and Heip are three estimators listed with error bars representing standard deviation (*n* = 3). Different letters indicate significant differences (LSD test, *p* < 0.05).

To further excavate the microbial potential, we conducted ozone exposure. During the low-level exposure (100~300 ppb), phyllospheric bacteria and fungi responded differently regarding the community structure. Copy numbers of 16S and ITS regions (per gram leaves) in Figure S2 and alpha diversity indexes in Figure 2 were combined for quantitative and qualitative analysis. A level of 16S rDNA concentrations presented a pronounced drop of about 43.6% and 72.1% compared to the corresponding control groups. Meanwhile, the OTU number and the Shannon index in Figure 2A remained almost unchanged, while Heip decreased from 0.099 to 0.031 after 10 d of exposure but subsequently returned to the original level. The trends suggested the phyllospheric bacteria experienced an abundance and homogeneity loss initially but acclimated to the low-level exposure within the experimental period. On the other hand, fungi experienced insignificant concentration change (Figure 2B), but at the cost of lower abundance (527~329 of OTU number after 28 d exposure), Shannon diversity (2.8~1.7 of Shannon index after 28 d exposure), and evenness (5.3~3.7 of Heip estimator after 10 d exposure). Unlike bacteria, fungi failed to sustain the community richness and diversity in the long run, despite the continuously stable ITS concentration. The above conclusions were consistent with other researches, where phospholipid fatty acid (PFLA) analysis suggested ozone exposure reduced total, bacterial, and fungal biomasses in the bulk soil [32], and fungi seemed more sensitive during the exposure [33]. However, findings in the soil might offer limited reference, since phenomena such as littering and nutrient distribution are inapplicable in the phyllosphere [17].

Microbial compositions were simultaneously altered during the ozone exposure. For bacterial phyla (Figure S5A), the relative abundance of Actinobacteriota increased from 58.7% to 77.0% (*p* < 0.05) after 10 d exposure, which showed the nevertheless unobvious difference (*p* > 0.05) between control (79.1%) and exposure groups (73.4%) after another 18 d treatment. Proteobacteria witnessed a similar trend (*p* < 0.05), suggesting low-level ozone stress caused limited influence. Notably, Firmicutes were significantly enhanced (*p* < 0.05) after 10 d. The compositions of fungi were straightforward, with only two dominant phyla, Ascomycota and Basidiomycota, and Ascomycota obtained higher dominance (75.3~96.2%, *p* = 0.05) as the experiment progressed. In general, the compositions at the phylum level presented insignificant changes regarding both bacteria and fungi under the ozone stress, while the sampling time surprisingly contributed to more variations. Similar observations were reported in an ozone exposure of orange trees, where environmental differences from chambers resulted in more significant effects than the ozone treatments [14]. Other critical determinants such as nutrient availability or meteorological conditions probably overrode ozone exposure.

**Figure 3:**
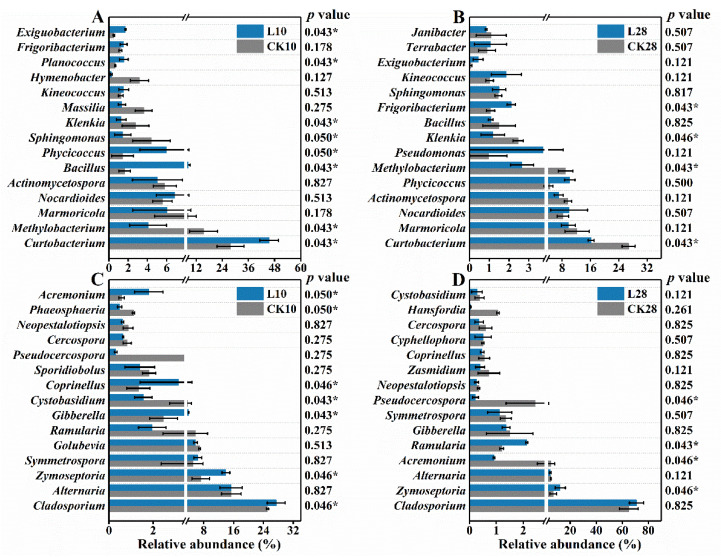
The microbial composition successions in the phyllosphere during the low-level ozone exposure. Bacteria compositional changes after 10 d (**A**) and 28 d (**B**) exposure and fungal compositional changes after 10 d (**C**) and 28 d exposure (**D**). Error bars represent the standard deviation of replicates (*n* = 3). *p* values were calculated by Mann–Whitney–Wilcoxon (MWW) test (* 0.01 < *p* ≤ 0.05).

At the genus level, dominant taxa generally sustained the edge. Relative abundance of the top 15 abundant bacterial genera is listed in Figure 3A,B to indicate the impacts of 10 d and 28 d ozone exposure, respectively. *Curtobacterium* was the most abundant genus throughout the experiment, which first increased from 27.8% to 45.5% and then fell from 26.8% to 16.2%. *Curtobacterium* species are mainly associated with the phyllosphere and play various ecological roles such as the causal agent [34] and endophytic symbionts [35]. The multifunctionality and endophytism might account for its resistance. *Methylobacterium* growth was inhibited during the exposure, suggesting its sensitivity to ozone, and *Masilia, Hymenobacter* presented a similar trend. *Hymenobacter* can acclimate to the urban environment and habitats with strong UV radiation. It showed the potential of utilizing fungal carbon [36], but these favoring traits seemed insufficient to combat the present ozone stress. By contrast, *Bacillus*, *Exiguobacterium*, *Phycicoccus*, and *Frigoribacterium* experienced a noticeable abundance rise of 6.9%, 1.2%, 5.6%, and 1.1%, respectively, after 10 d or 28 d exposure. The versatile genus *Exiguobacterium* can potentially enhance plant growth and agricultural production [37]. *Phycicoccus* species possibly contain putative genes for alleviating stresses in plant-associated environments [38]. Their resistance could be partially ascribed to such advantages. 

Fungal compositions are shown in Figure 3C,D, with insignificant alterations. The predominant genus, *Cladosporium*, further strengthened and peaked at 70.9% after 28 d. Notably, the enhancement was attributed to factors other than the ozone input, evidenced by the simultaneous abundance rise in the control groups. *Cladosporium* includes common endophytes, plant pathogens and fungal hyperparasites [39], while certain species are proved as potential biocontrol agents for plant diseases [40]. *Zymoseptoria*, *Gibberella*, *Acremonium*, and *Ramularia* presented trends upward to different extents, while the growth of genera *Golubevia*, *Cystobasidium*, *Phaeosphaeria*, *Pseudocercospora* and *Hansfordia* were significantly restricted, *Hansfordia* (almost extinct) in particular. *Zymoseptoria tritici* is a hemibiotrophic pathogen that causes Septoria leaf blotch in wheat [41]. *Golubevia* species are biocontrol agents of plant-pathogen [42], the shrink of which might favor pathogenic invasions. *Pseudocercospora* is a plant pathogen causing leaf spots [43] and was almost eliminated during the ozone exposure. *Hansfordia* species were found parasitizing tomato leaf mold and *Cladosporium cladosporioides* [44]. Most core fungal taxa were related to plant diseases, either causal or biocontrol agents, and parasitism, likely to be intensified by the ozone surplus and the sealed condition. Such varying sensitivity among species could lead to potential biological control loss or enhancement in the phyllosphere.

### 3.3. (Ultra)High-Level Ozone Exposure

**Figure 4:**
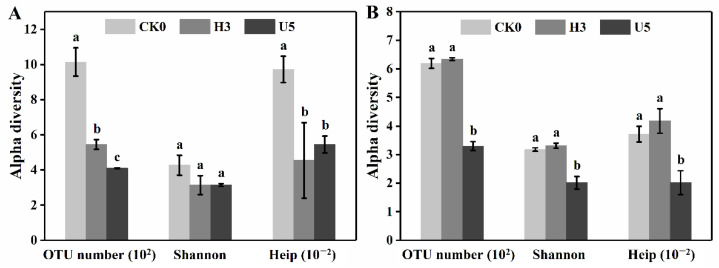
Alpha diversity changes of the bacterial (**A**) and fungal (**B**) community in the phyllosphere during the (ultra)high-level ozone exposure. OTU numbers, Shannon and Heip, are shown with error bars representing standard deviation (*n* = 3). Different letters indicate significant differences (LSD test, *p* < 0.05).

We then conducted short-term high-level and ultrahigh-level ozone exposure on *E. japonicus*. The alterations in the species diversity are illustrated in Figure 4. Exposure to the high-level ozone induced a converse pattern to the low-level one. Phyllosphere fungi barely changed, while bacteria’s richness, diversity, and evenness decreased by 46.2%, 26.6%, and 53.3%, respectively. By contrast, 5 d exposure to the ultrahigh-level ozone damaged bacterial and fungal diversity, especially the richness and evenness. The bacterial community in the phyllosphere was highly sensitive to the acute ozone stress compared to fungi. While the fungal community was also considerably deteriorated by the ultrahigh-level ozone, despite its tolerance to the high-level ozone exposure. It is not surprising to observe community structure deterioration since massive reports evidenced the lethal effect during direct exposure to high-level ozone [45,46]. The notable fungal tolerance during the high-level exposure might be attributed to the relatively shorter duration.

Based on the compositions at the phylum level in Figure S6A, except that Actinobacteriota obtained a higher abundance from 29.7% to 72.4% (*p* < 0.05), other phyla experienced varying degrees of abundance drop. The changing patterns during the ultrahigh-level exposure were similar. Most Actinobacteriota are saprophytes, with a limited proportion being plant pathogenic [47]. Phyllosphere fungi consist of Ascomycota and Basidiomycota, and Ascomycota increased by 21.3% (*p* < 0.05) and 27.0% (*p* < 0.05) during the 3 d high-level and 5 d ultrahigh-level ozone exposure, respectively. Ascomycota is the most diverse fungal group, living by various nutritional modes [48] and shows remarkable tolerance to external perturbation.

**Figure 5:**
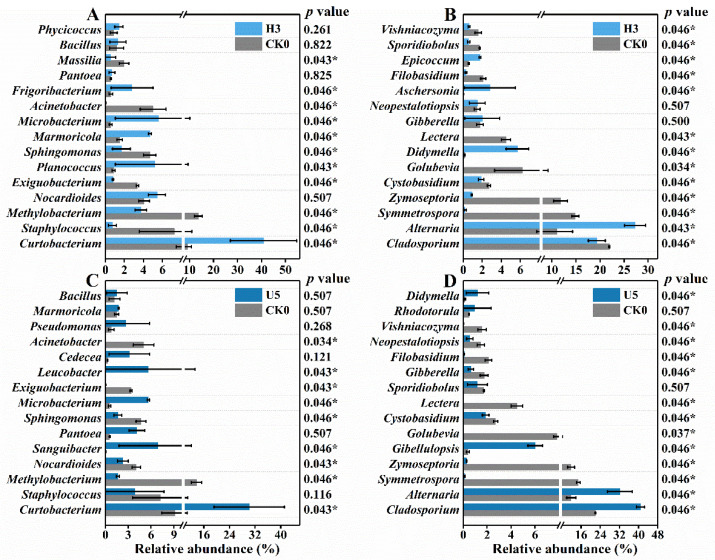
The microbial successions in the phyllosphere during the (ultra)high-level ozone exposure. High-level exposure: bacterial (**A**) and fungal (**B**) community compositions; ultrahigh-level exposure: bacterial (**C**) and fungal (**D**) community compositions. Error bars represent the standard deviation of replicates (*n* = 3). *p* values were calculated by Mann–Whitney–Wilcoxon (MWW) test (* 0.01 < *p* ≤ 0.05).

The exposure induced distinct changes at the genus level. During the high-level exposure (Figure 5A,B), nearly half of the top 15 abundant bacterial genera were enhanced, with the remaining reduced, resulting in an insignificant change in the bacterial diversity. Remarkably, the relative abundance of *Curtobacterium* and *Marmoricola* rose from 9.1% to 40.8% and from 1.5% to 4.7%, respectively. Conversely, genera *Methylobacterium*, *Staphylococcus*, *Exiguobacterium*, and *Acinetobacter* witnessed an abundance drop of 10.0%, 6.5%, 2.6%, and 5.0%, respectively. Among them, *Acinetobacter* almost went extinct under the ozone stress. *Marmoricola* presents great tolerance under long-term haze pollution [49]. *Staphylococcus* was identified as a human pathogen through wound infections, pneumonia, etc. [50]. Intriguingly, topical ozone therapy was employed to alleviate *Staphylococcus*-induced atopic dermatitis, where *Acinetobacter* might be probiotic [51]. The inconsistency with the present observation might correlate to the ozone intensity.

The responses of phyllospheric fungi were more apparent, as *Alternaria* outcompeted *Cladosporium* and dominated the phyllosphere with a relative abundance of 27.3%. Similarly, *Didymella*, *Aschersonia*, and *Epicoccum* were increased by 5.5%, 2.8%, and 1.2%, respectively. Meanwhile, most genera were severely inhibited and nearly extinct by the ozone stress, represented by *Symmetrospora*, *Zymoseptoria*, *Golubevia*, and *Lectera*, indicating their high sensitivity. *Didymella* contains several serious plant pathogens and endophytic and saprobic species on crops [52]. *Aschersonia* species have shown potential as pest-control agents [53]. *Epicoccum* species may act as plant pathogens, but some endophytic species have been associated with biological control [54]. These core taxa again evidenced that ozone possibly intensified the antagonism related to plant diseases.

We subsequently elevated the exposure level to 10,000 ppb, and different changing patterns were observed in bacterial (Figure 5C) and fungal (Figure 5D) compositions in the phyllosphere. For instance, bacterial genera *Sanguibacter*, *Pantoea*, *Leucobacter*, and *Cedecea* surprisingly benefitted from the ozone input with an abundance increase of 6.8%, 3.6%, 5.6%, and 2.8%, respectively. *Pantoea* species were first recognized as plant pathogens but subsequently linked to human infections and biocontrol products. *Leucobacter* was once isolated from the potato phyllosphere, but the related traits and interactions remained unclear [55]. The fungal community after 5 d ultrahigh-level exposure was dominated by *Cladosporium* (40.8%) and *Alternaria* (32.2%), leaving limited resources for other populations and leading to lower diversity and richness (Figure 5B). Notably, *Cladosporium* gained more advantage under the ultra-high ozone stress than the high-level exposure, and *Gibellulopsis* manifested a similar upward trend, from 0.4% to 6.0%. On the other hand, several resistant genera, represented by *Didymella* and *Aschersonia*, were severely suppressed by the elevated exposure intensity, suggesting the duality of ozone and the optimal concentration. 

**Figure 6:**
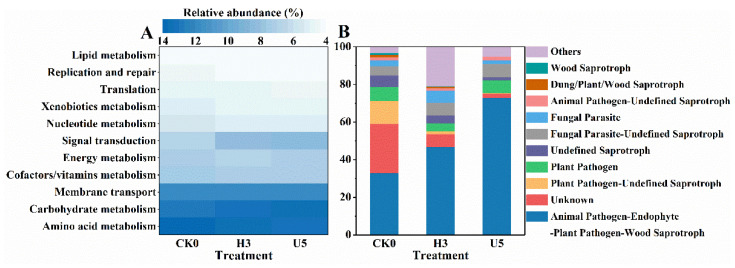
Functional predictions and comparisons among different treatments: (**A**) relative abundance (%) of main level 2 Kyoto Encyclopedia of Genes and Genomes (KEGG) orthology groups based on Tax4Fun; (**B**) relative abundance (%) of major fungal guilds screened by FUNGuild. Values were presented as the mean (*n* = 3).

The (ultra)high exposure inevitably altered microbial functions. KEGG-based functional annotation (Figure 6A) showed that bacterial functions mainly consisted of amino acid metabolism (13.1% ± 0.4%, mean ± SD, *n* = 9), carbohydrate metabolism (12.9 ± 0.2%), and membrane transport (11.4% ± 0.1%). These fundamental pathways were reported to facilitate energy supply, maintain cellular functions [56], and mount defensive strategies under external stress [57]. Insignificant change in bacterial functions was observed during the exposure, except for active signal transduction after high-level (7.7%) and ultrahigh-level (8.1%) compared to that in the natural phyllosphere (6.6%). Signal transduction is the process in which the binding of an extracellular messenger to the cell surface receptor is translated into changes for the cell to respond [58]. It is dominated by a two-component signal transduction system via cross-regulation, biofilm formation, etc. [59], stimulated by the ozone stress. Conversely, remarkable changes in fungal guilds were presented during the ozone exposure (Figure 6B). Animal and plant pathogens surged under stress, while saprotrophs were primarily reduced, indicating a pathogen predominance and deteriorated plant health.

## 4. Conclusions

In the present study, we tested the phylloremediation efficiency of *E. japonicus* and proved it a satisfactory plant material. The low-level ozone exposure (156 ± 20 ppb) imposed insignificant effects on the phyllosphere microbiome under the interference of other factors, especially the plant growth period. Bacteria and fungi presented different responsive patterns. Although fungi failed to sustain the community richness and diversity, the abundance remained unchanged, while bacteria manifested an opposite trend. A high exposure level up to 5000~10,000 ppb significantly changed the bacterial and fungal community within a short period, where extremely tolerant species were highlighted, including bacterial genera (*Curtobacterium*, *Marmoricola*, and *Microbacterium*) and fungal genera (*Cladosporium* and *Alternaria*). Most core fungal taxa were related to plant disease causal (e.g., *Zymoseptoria*) and biocontrol agents (e.g., *Golubevia*), forming an antagonistic relationship, possibly influencing the plant health and ozone uptake. The functional analysis suggested that the bacterial defense mechanism might relate to signal transduction and pathogenic fungi obtained a predominance under the ozone stress, posing a threat to the plant health. Our results are particularly relevant considering the ozone threats and the urgent need for a sustainable solution. The detailed reactions between the phyllospheric microbiota and ozone require further data at the gene level.

## Data Availability

Not applicable.

## Supplementary Materials

The following supporting information can be downloaded at: https://www.mdpi.com/article/10.3390/microorganisms10040680/s1, Table S1: The properties of the tested plants in the ozone remediation and exposure experiments; Figure S1: The schematic diagram of the experimental chamber; Table S2: The specification of each component in the apparatus; Figure S2: Ozone concentrations in chambers and the synchronous background level during the 28-d low-level exposure; Figure S3: T (°C) and RH (%) in chambers during the 28-d low-level exposure; Figure S4: 16S and ITS concentrations in per gram leaf during the low-level ozone exposure; Figure S5: Relative abundance changes of bacterial (A) and fungal (B) phyla in the phyllosphere during the low-level ozone exposure; Figure S6: Relative abundance changes of bacterial (A) and fungal (B) phyla in the phyllosphere during the (ultra) high-level ozone exposure.
