# Supplementary material for: Responses of Phyllosphere Microbiome to Ozone Stress: Abundance, Community Compositions and Functions
Source: Microorganisms. 2022 Mar 22;10(4):680. doi: 10.3390/microorganisms10040680 (PMC9024792; doi:10.3390/microorganisms10040680)
Supplement: Supplementary file 1 [file microorganisms-10-00680-s001.zip › microorganisms-1626016-supplementary.pdf]

Responses of phyllosphere microbiome to ozone stress: abundance, community compositions and functions

Jiayu Liu [a,1,2], Manjiao Song [a,2,3], Xinyuan Wei [2], Huanzhen Zhang [1], Zhihui Bai [2,3,4,*], Xuliang Zhuang [2,3,5,*]

[a] These authors contribute equally to this paper.

[1] Beijing Key Laboratory of Water Resources and Environment Engineering, School of Water Resources and Environment, China University of Geosciences (Beijing), Beijing, 100083, China.

[2] Key Laboratory of Environmental Biotechnology, Research Center for Eco-Environmental Sciences, Chinese Academy of Sciences, Beijing, 100085, China.

[3] Sino-Danish College, University of Chinese Academy of Sciences, Beijing, 100049, China.

[4] Xiongan Institute of Innovation, Xiongan New Area, 071000, China.

[5] Institute of Tibetan Plateau Research, Chinese Academy of Sciences, Beijing, 100101, China.

[*]Corresponding authors.

E-mail addresses: zhbai@rcees.ac.cn (Z. Bai), xlzhuang@rcees.ac.cn (X. Zhuang)

Table S1. The properties of the tested plants in the ozone remediation and exposure experiments. D, H stand for the projected area of the canopy and the above-ground height, respectively.

Species	Purpose	D (cm)	H (cm)
Euonymus japonicus	Phylloremediation	20~25	20~25
	Exposure	45~50	60~70

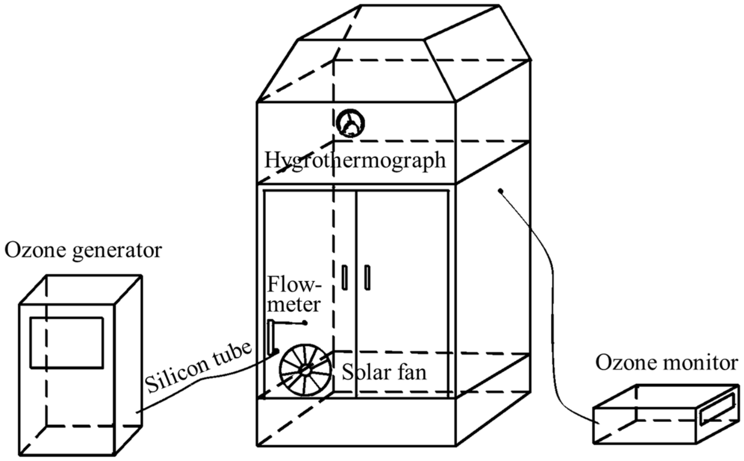

Figure S1. The schematic diagram of the experimental chamber

Table S2 The specification of each component in the apparatus

Components	Specification
Outer membrane	Teflon
Solar fan	6V, 3W
Hygrothermograph	Deli, China, T: 0~50 °C, RH: 0~100%
Flowmeter	LZB-3WB, 0.15-1.5 L·min^{-1}
Portable ozone generator	KWNT-CYF2, BEIJING CONVENIENT ENVIROTECHCO.,LTD, China
Ozone monitor	Model 106-L, 2B Technologies, USA

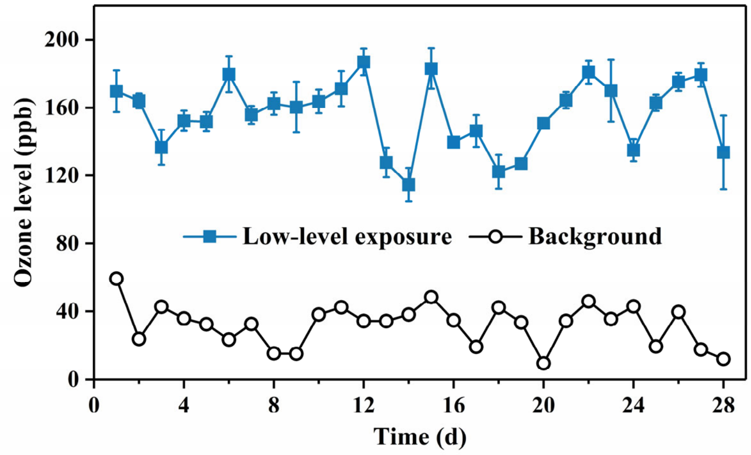

Figure S2. Ozone concentrations in chambers and the synchronous background level during the 28-d low-level exposure. Error bars represent standard deviation (n=3).

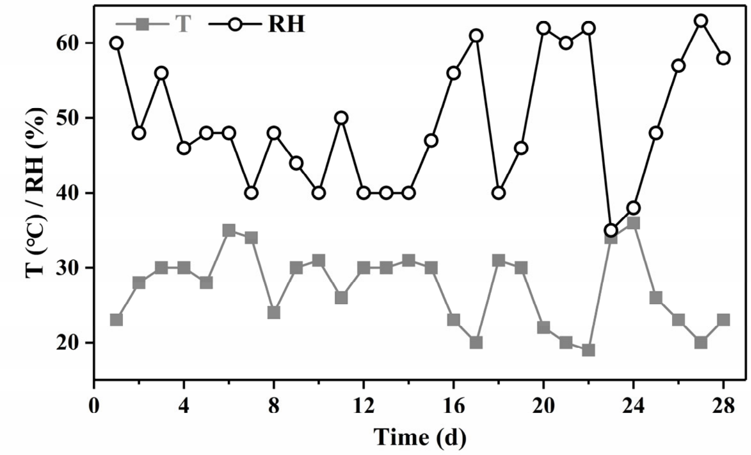

Figure S3. T (℃) and RH (%) in chambers during the 28-d low-level exposure

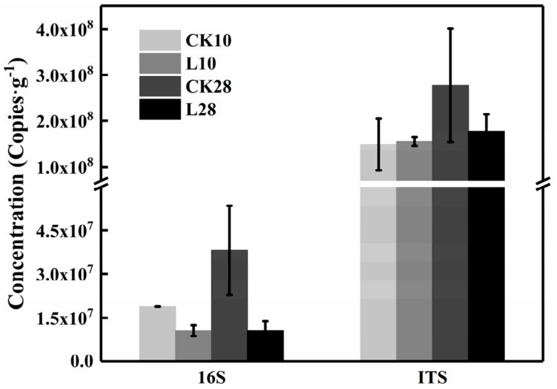

Figure S4. 16S and ITS concentrations in per gram leaf during the low-level ozone exposure. Error bars represent standard deviation (n=3).

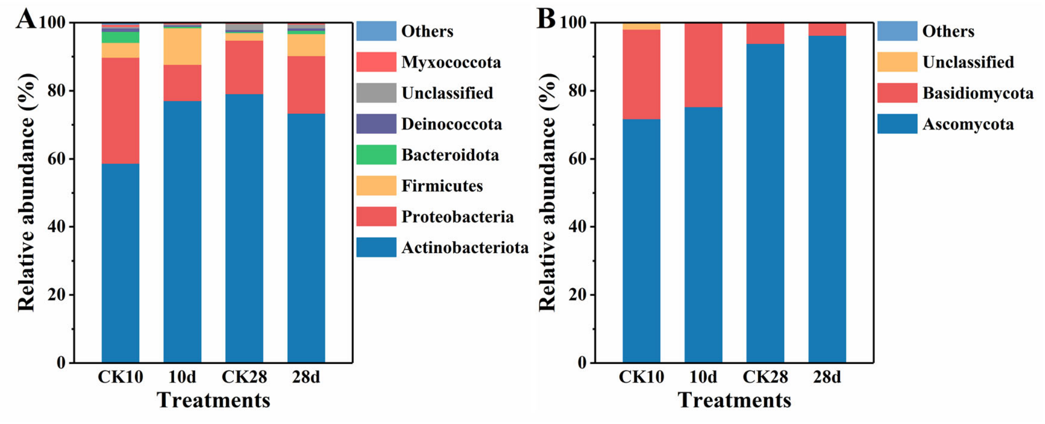

Figure S5. Relative abundance changes of bacterial (A) and fungal (B) phyla in the phyllosphere during the low-level ozone exposure. Values were presented as the average (n=3).

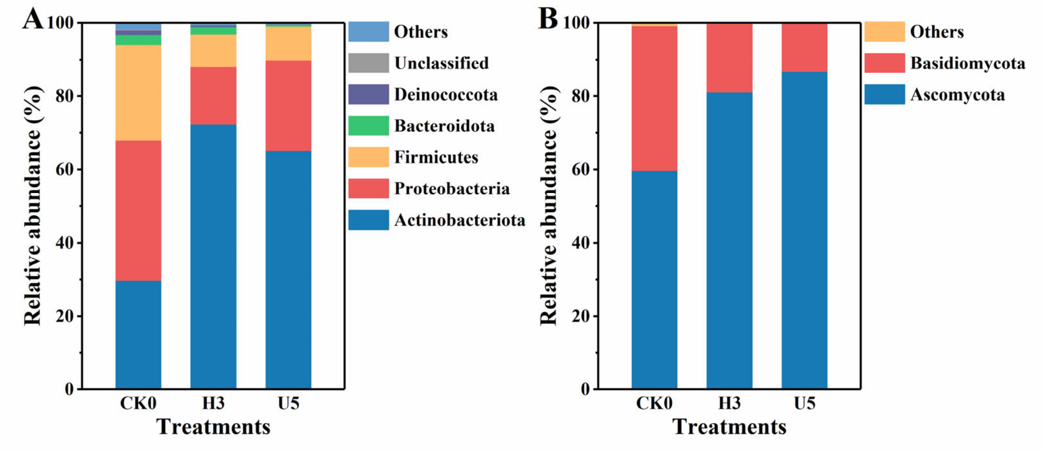

Figure S6. Relative abundance changes of bacterial (A) and fungal (B) phyla in the phyllosphere during the (ultra) high-level ozone exposure. Values were presented as the average (n=3).
